# Intraoperative 3D Comparison of Round and Anatomical Breast Implants: Dispelling a Myth

**DOI:** 10.3390/jcm11010149

**Published:** 2021-12-28

**Authors:** Luisa Lotter, Isabel Zucal, Vanessa Brébant, Norbert Heine, Robin Hartmann, Karolina Mueller, Lukas Prantl, Daniel Schiltz

**Affiliations:** 1 Department of Plastic, Hand- and Reconstructive Surgery, University Hospital Regensburg, 93053 Regensburg, Germany; luisa.lotter@googlemail.com (L.L.); isabel.zucal@icloud.com (I.Z.); vanessa.brebant@ukr.de (V.B.); norbert.heine@ukr.de (N.H.); robin-hartmann@t-online.de (R.H.); lukas.prantl@ukr.de (L.P.); 2 Center for Clinical Studies, University Hospital Regensburg, 93053 Regensburg, Germany; karolina.mueller@ukr.de; 3 Department of Plastic and Aesthetic Surgery, Hand Surgery, Helios Hospital Emil von Behring, Walterhöferstraße 11, 14165 Berlin, Germany

**Keywords:** breast augmentation, breast implant, 3D-volumetry, 3D measurement, 3D scan

## Abstract

Background: Thanks to 3D imaging, it is possible to measure the influence of different parameters on breast augmentation. In this study, we compare the effect of different shapes and sizes of breast implants on the topography of the resulting breast. Furthermore, the impact of different breast implants on inter-landmark distances and on changes of the nipple position was assessed. Methods: This interventional prospective study was carried out on 10 female patients after collecting informed consent. 3D scans of the native and augmented breasts were performed intraoperatively with small, medium, and large sizes of both anatomical and round implants, resulting in a total of *n* = 130 single breast scans. These scans were analyzed for topographic shift quantification, nipple migration, and inter-landmark distances of the breast. Results: Implant size, but not implant shape leads to significant topographic shifts of the breast (*p* < 0.001 and *p* = 0.900, respectively). Both round and anatomical implants lead to a significantly higher volumetric increase in the upper quadrants compared to the lower quadrants (*p* < 0.001). Nipple migration into the superomedial quadrant was seen in about 90% of augmentations. No evident differences in inter-landmark distances were observed when round and anatomical implants of different sizes were compared. Conclusions: Implant size rather than shape influences the postoperative aesthetic results. No significant difference in topographic shift was found comparing round and anatomical implants, suggesting that both implant shapes result in comparable aesthetic outcomes.

## 1. Introduction

In the past decade, the introduction of new technologies for the digital measurement of the surface of bodies in three-dimensions (3D) has offered new possibilities for numerically recording the parameters which influence the shape of the female breast in the course of breast augmentation. These recordings support the surgeons in their planning and decision-making [1,2,3,4,5,6]. The choice of the appropriate implant has been discussed in several studies as one of the most important factors affecting the postoperative appearance of the breasts after augmentation [7,8,9]. Due to the lack of comparison between different implant shapes and volumes in the same patient [7,8] and the limited use of accurate measurements [9] obtained from photography, which can only reproduce the topography of the breast to a limited extent, data are not sufficient to enable an evidence-based choice of the most suitable implant. The primary goal of this study was to determine the effects of different implant shapes (round vs. anatomical) and volumes on the topography of the female breast in the same patient and to assess if there is a significant difference in the augmented breast topography depending on the breast implant size or shape. Secondary goals were: 1. The analysis of how the shape and volume of the breast implants determine the changes of inter-landmark distances of the breast (jugulum-nipple distance, nipple-inframammary fold (IMF) distance, etc.); 2. the assessment of how changes of the nipple position depend on the shape and volume of the breast implant.

## 2. Materials and Methods

The study was planned as an interventional prospective study of ten female volunteers who all gave informed consent. All patients were operated between February and August 2019 at Caritas hospital St. Josef, Regensburg, Germany. Previous breast operations, epilepsy, or breast ptosis grade 3 (Regnault) were exclusion criteria. The study was approved by the Ethics Committee of the Universities of Regensburg (reference numbers: 18-885-101 and 18-1030-101; clinical trial number: DRKS00022497) and planned in accordance with the Helsinki Declaration of 1975.

### 2.1. General Procedure

All surgical procedures were carried out in a supine position with the arms outstretched at 45° under general anesthesia. To control the implant position and generate the scans, once the pocked was prepared, patients were brought into beach chair position (upper body up to 55°). After disinfection and sterile covering, 3D scans of the breasts were performed intraoperatively, without (“native”) and with (“implanted”) breast implants, after preparation of the implant pocket (submuscular in eight and subglandular plane in two patients who were very athletic and animation deformity was feared). All patients were operated by the same surgeon (Prof. Lukas Prantl). Incision was five times in the inframammary fold (5–8 cm length) and five times periareolar. All scans were performed in the same position (55° upright, measured using a goniometer and a level). In total, six pairs of implants, differing in shape (round/anatomical) and volume (small/medium/large) were inserted and measured after temporary wound closure. In seven patients, all six different implants were inserted, resulting in 49 scans (native scans included), in two patients, only four study implants could be used (= 8 implanted + 2 native scans), and in one patient, one study implant was missing (= 5 implanted + 1 native scan). This resulted in 65 3D breast scans and a total count of *n* = 130 single-breast images. These were processed and evaluated using the scanner compatible Artec Studio 12 Professional software (Artec, Luxembourg). To assess the influence of the respective implants on the properties of the breasts, the “topographic shift” (TS) was determined. The TS describes the estimated mean distance of the “native” and “implanted” 3D-scan of the same breast in mm by laying one on top of the other [10]. The TS, inter-landmark distances and changes of the nipple position were determined. The right and left breasts were considered separately as they often have substantial differences in shape and size despite belonging to the same patient.

### 2.2. Implant Selection, 3D Scan, and Measurements

**Figure 1:**
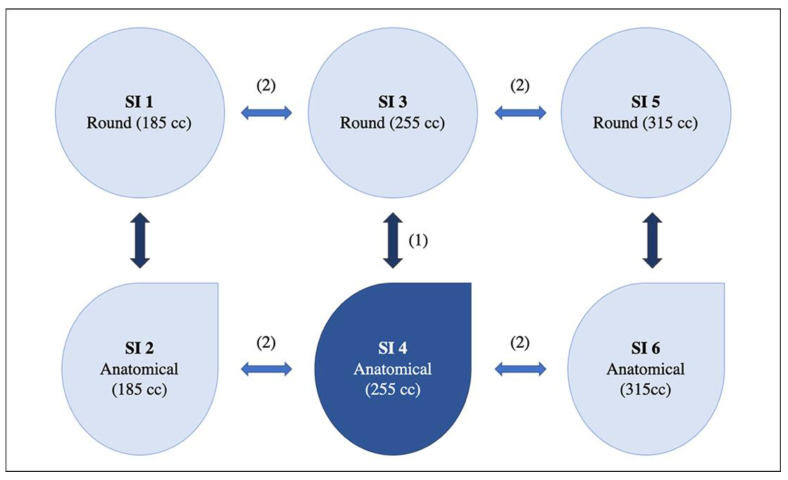
Schematic example of implant selection. First, the definitive implant was chosen during preoperative consultation. Thereafter, the implant of the contrary shape and same volume was chosen and finally, the smaller and bigger pairs of implants were chosen for each shape. In this example, an anatomical implant with 255 cc of volume was chosen as definitive implant. SI 1, 3, and 5 were round implants and SI 2, 4, and 6 were anatomical implants. SI = study implant. Dark blue: specific implant selected in the preoperative consultation. (1) Selection of the round correlate with same base +/− 5 mm, volume and projection. (2) Selection of the smaller/larger projection with same shape/base.

All implants used in this study were textured, definitive implants from Polytech, POLYtxt Sublime Line, Germany. No test implants were used. Six different implants, differing in shape (anatomical/round) and volume (small/medium/large), with constant base within the respective shape, were selected for each patient according to the following algorithm (Figure 1): The first implant was the one chosen to be the final implant during preoperative consultation. The second one had the same base +/− 5 mm according to the manufacturer’s specifications, but the opposite shape, allowing for a direct comparison between the anatomical and round shapes. Based on this pair of implants, the smallest and largest available sizes of each shape were selected while maintaining the base. Due to the manufacturer’s (Polytech, POLYtxt Sublime Line) volumes for different shapes, some volume mismatch between the two shapes had to be tolerated for some pairs of implants (difference range: 5–10 mL). The study implants (SI) were classified into six groups: SI 1–6. In order to rule out the possibility that the breast tissue may expand itself due to a large volume implant, leading to false measurements of smaller implants, the order of implant insertions was from the smallest to the largest in volumetric terms.

A hand-held and mobile 3D scanner (Eva, Artec, Luxemburg) was used to make the intraoperative 3D scans. Scans were processed on a computer by the Artec Studio 12 software (Artec, Luxembourg). After processing, a 3D model of each pair of implants was available. Using this model, various objective calculations could be carried out. Evidence of a valid and reproducible analysis of the breast contour and its volumetry using this or similar methods has already been shown several times [10,11,12,13,14,15].

### 2.3. Topographic Shift (TS) over the Four Breast Quadrants

The native scan without implant served as a reference and the Artec Studio 12 software (Artec, Luxembourg) automatically detected the appropriate landmarks. The mean topographic shift (in mm) was calculated for each implant size and shape in all breast quadrants (quadrant I = superolateral, quadrant II = superomedial, quadrant III = inferomedial, and quadrant IV = inferolateral quadrant). This method has been precisely described and published by our research team [10].

### 2.4. Inter-Landmark Distances

**Figure 2:**
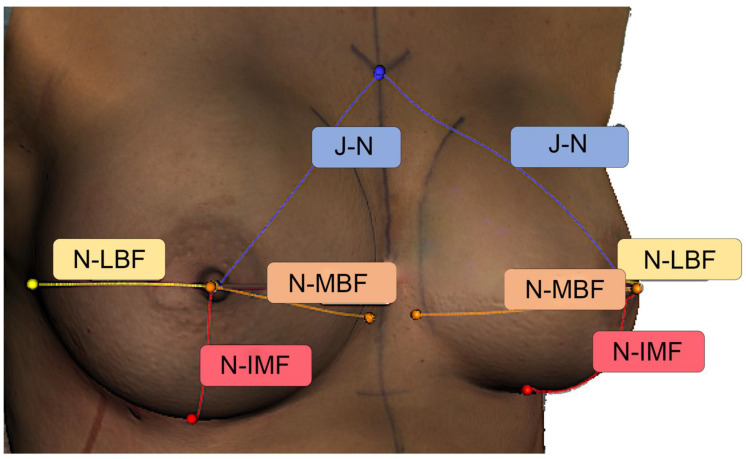
Anthropometric measurements in an intraoperative 3D scan using the Artec Studio 12 software (Artec, Luxembourg). Here we provide an example of 3D measurements of the breast. N-LBF = nipple—lateral breast fold distance, N-IMF = nipple—inframammary fold distance, N-MBF = nipple—medial breast fold distance, J-N = jugulum—nipple distance. Distances are indicated in millimeters with matching colors.

Based on past studies [16,17] and the resulting literature recommendations, the following distances between anatomical landmarks were measured (in mm): jugulum-nipple, nipple-inframammary fold (IMF), nipple-lateral breast fold, nipple-medial breast fold (Figure 2). The distances were measured in all ten patients on the right and left breast for all SI.

### 2.5. Position Change of the Nipple

An implant insertion affects the position of the nipple. The movement of the nipple, and in which of the four quadrants it moved was determined by comparing its position in the “native” and “implanted” scans. The “native” scan of each breast served as the reference for all measurements. If the nipple moved in two quadrants at the same time (borderline), preference was given to the quadrant in which most of the nipple moved.

### 2.6. Statistics

A mixed linear model was created for the analysis of the TS. Parameter estimation was carried out using the method of maximum likelihood and compound symmetry was used as a repeated covariance type. Twenty breasts with twenty-four measurements each (six implants × four quadrants), were analyzed and compared to the native scan. The TS was used as the dependent variable. The fixed main effects were shape (round vs. anatomical), implant size (small vs. medium vs. large), and quadrant (I vs. II vs. III vs. IV). The fixed interaction effects were implant size/quadrant, implant size/shape, and quadrant/shape. There was no adjustment for multiple measurements.

To calculate the differences in inter-landmark distances, the following formula was used: distance X_round implant_—distance X_anatomical implant_. Means of differences were calculated for small (SI 1 and 2), medium (SI 3 and 4), and large (SI 5 and 6) implants.

A frequency table was created for each shape in order to consider the change of position of the nipple separately for round and anatomical implants. The McNemar test was used to test for differences between round and anatomical implants.

In order to indicate statistical significance, all tests were carried out on both sides with a significance level of *p* ≤ 0.05 with the software SPSS version 26.0 for Windows (SPSS Inc., Chicago, IL, USA).

## 3. Results

Patients had a median age of 32 (22–49) years, a median weight of 63 (54–76) kg, and a median BMI of 22 (19–26) kg/m^2^. Five patients had no breast ptosis, three had 1st degree ptosis according to Regnault’s classification, and two had 2nd degree ptosis. An overview of demographic data is provided in Table 1. The used implants and the average, minimum, and maximum volumes of the SI 1–6 are listed in Table 2. In one case, the largest pair of implant size calculated using the selection algorithm (SI 5 and 6) would have increased the risk of complications by an unacceptable margin, so the decision was made not to use these implants. In another patient, the smallest sizes (SI 1 and 2) were not available and in a further patient, SI 6 was not available. Comparing the means of the volumes of the SI 1 and 2 (small round vs. anatomical), 3 and 4 (middle round vs. anatomical), 5 and 6 (large round vs. anatomical), there were relatively small differences: −0.6 cc (−0.3%, SI 1 vs. 2), −2.0 cc (−0.7%, SI 3 vs. 4), and 9.7 cc (2.7%, SI 5 vs. 6). This was due to the implant characteristics determined by the manufacturer.

### 3.1. Topographic Shift (TS) over the Four Breast Quadrants

#### 3.1.1. Main Effect: Shape (Round vs. Anatomical)

The mean difference of the TS between the round and anatomical shapes (all implants) was not significant (*p* = 0.900). Breast augmentations with anatomical implants led to a mean TS 0.6% (0.04 mm, 95% CI = −2.15–2.23 mm) higher than those with round implants.

#### 3.1.2. Main Effect: Size (Small vs. Medium vs. Large)

The mean TS differed significantly with increasing implant size (*p* < 0.001). With small sized implants, the mean TS was 23.3% (1.24 mm, 95% CI = −1.02–3.50 mm) smaller than with the medium size. With medium sized implants the mean TS was 20.5% (1.34 mm, 95% CI = −0.93–3.60 mm) smaller than with large size.

#### 3.1.3. Main Effect: Quadrant (I vs. II vs. III vs. IV)

Significant changes of the TS (*p* < 0.01). The smallest volumetric effect of implant insertion was observed in quadrant IV, followed by quadrant III. In quadrants I and II there was a significantly higher increase in volume. Hence, the principal volumetric effect of breast implants was mainly located in the upper breast quadrants (Table 3).

#### 3.1.4. Interaction: Implant Size × Shape

**Figure 3:**
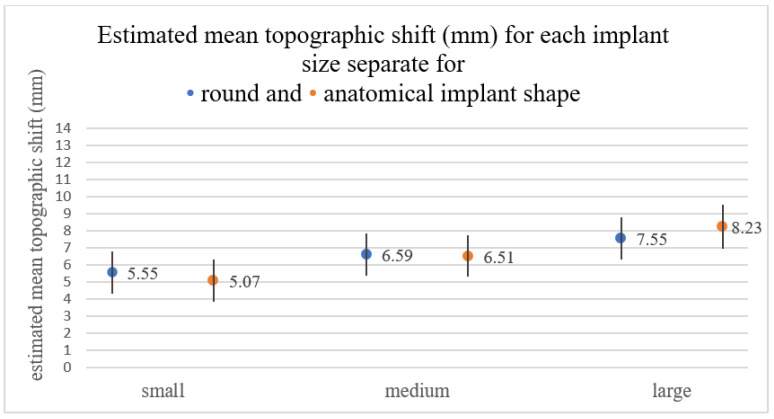
Estimated mean TS for each implant size (small/medium/large) in round and anatomical shaped implants. No significant difference was observed between round and anatomical shape in same-sized implants. However, in large implants, a bigger difference between implant shapes was observed (round: 7.55; anatomical: 8.23) compared to the medium and small sizes.

Within implant sizes, no difference was observed between round and anatomical implants (*p* = 0.323). In large implants, a bigger difference between implant shapes was observed compared to the medium and small sizes, but it was not significant (*p* = 0.227) (Figure 3).

#### 3.1.5. Interaction: Implant Size × Quadrant

**Figure 4:**
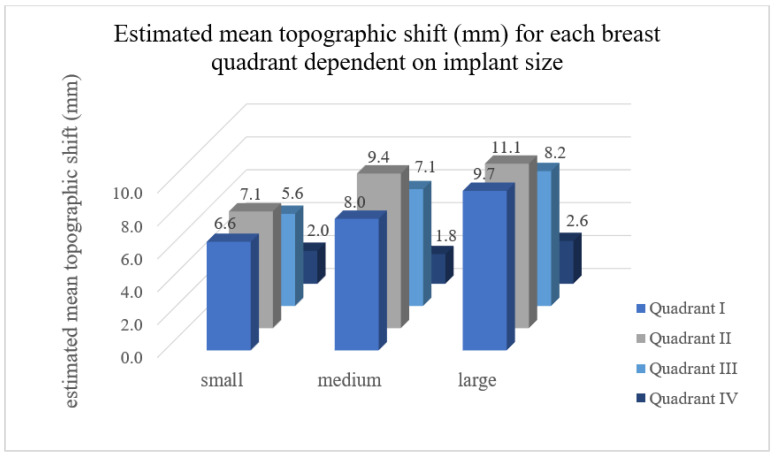
Estimated mean TS for each breast quadrant dependent on implant size. Quadrant I = superolateral, quadrant II = superomedial, quadrant III = inferomedial and quadrant IV = inferolateral breast quadrant. In quadrant I, II, and III the increase of the TS correlated with the implant size (round and anatomical shape). In quadrant IV, no significant difference in TS was observed when comparing between implant sizes.

In the post-hoc tests it was found that in quadrant I, large-sized implants resulted in a significantly higher mean TS compared to the small implants (*p* < 0.001) and medium implants (*p* = 0.022). No significant difference was found between small and medium sized implants (*p* = 0.065). In quadrant II, large implants resulted in the highest TS, followed by the medium and finally small. In this quadrant, the increase of the TS correlated with the implant size. In quadrant III, small implants resulted in a significantly lower volumetric effect compared with medium (*p* = 0.049) and large implants (*p* = 0.001). No significant difference in TS was found when comparing medium and large implants. Finally, in quadrant IV, no significant difference in TS was observed when comparing between implant sizes (Figure 4).

#### 3.1.6. Interaction: Quadrant × Shape

**Figure 5:**
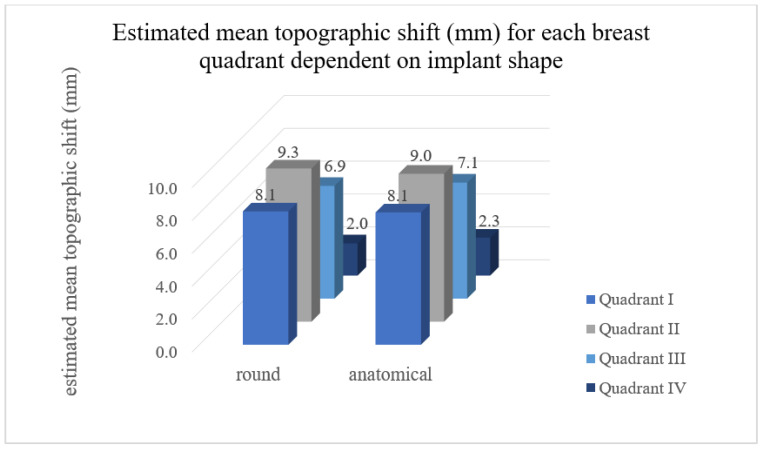
Estimated mean TS for each breast quadrant dependent on implant shape. Quadrant I = superolateral, quadrant II = superomedial, quadrant III = inferomedial and quadrant IV = inferolateral breast quadrant. No significant difference was observed between the shapes “round” and “anatomic” concerning TS in all quadrants.

**Figure 6:**
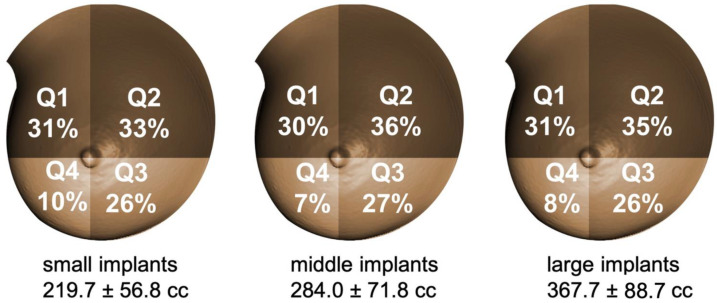
Percentual distribution of TS among quadrants with different implant sizes. Q1= superolateral, Q2 = superomedial, Q3 = inferomedial and Q4 = inferolateral breast quadrant. Comparable topographic breast quadrant changes were found in all implant sizes and shapes. More than 60% of TS was in the upper quadrants for both anatomical and round implants of each size.

No significant interaction was observed between shapes and quadrants (*p* = 0.876). The distribution of the TS between quadrants was comparable when comparing the two implant shapes (Figure 5). A maximal difference of 2% was found in quadrants II and IV. More than 60% of the increase of the TS was in quadrants I and II with both implant shapes and with all sizes (Figure 6).

### 3.2. Inter-Landmark Distances

The jugulum-nipple distance had a maximal variability of 3.1 mm within implants. No constant increase of the mean jugulum-nipple distance could be observed with increasing volume in anatomical implants (SI 2, 4, and 6), whereas it was observed in round implants (SI 1, 3, and 5). The mean nipple-IMF distance increased with bigger implant size in both round and anatomical implants. When comparing implants of the same volume size, the biggest difference in nipple-IMF distance (2.8 mm) was seen between SI 5 and 6. When comparing small (implant 1 vs. 2) and medium-sized implants (SI 3 vs. 4), it was observed that the mean difference between inter-landmark distances was ≤1 mm. For the large-sized implants (SI 5 vs. 6), a mean difference of >1 mm was found in the jugulum-nipple and nipple-IMF fold distance. The mean nipple-lateral and nipple-medial breast fold distance differed by ≤1 mm (Table 4).

### 3.3. Position Change of the Nipple

**Figure 7:**
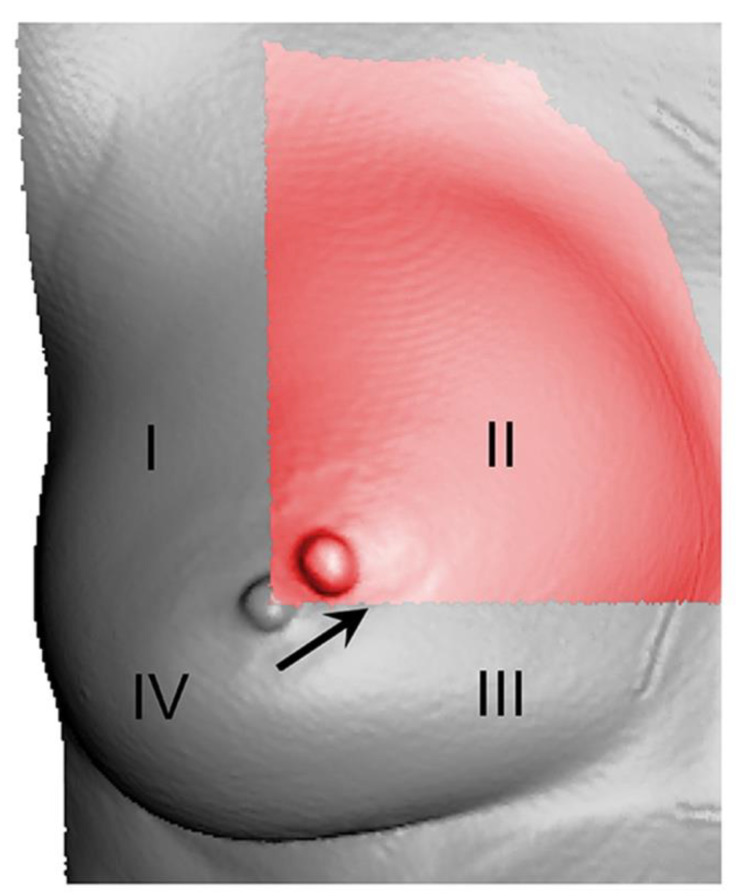
Nipple position change. I = superolateral, II = superomedial, III = inferomedial and IV = inferolateral breast quadrant. This is an example on how the nipple moves into quadrant II after augmentation in a 3D scan. Native scan = grey, scan of the augmented breast with anatomical implant of size 320 cc = red.

According to McNemar’s test, no significant difference between the round and anatomical implants was found comparing the nipple position change (*p* = 0.721). In 90.7% of augmentations with round and 88.9% of augmentations with anatomical implants there was a migration of the nipple into quadrant II (Figure 7). Overlapping nipple position among quadrants was seen in 48 of 54 cases comparing between the round and anatomical implants (twice in the superolateral quadrant, forty-six times in the superomedial quadrant and none in the inferomedial quadrant). No migration into quadrant IV was observed with either implant type (Table 5).

## 4. Discussion

The myth of achieving a more natural result using anatomical implants instead of round implants in breast augmentation was already dispelled in several studies [8,9,18,19]. However, this myth persists today [20,21,22]. In the past, in postoperative evaluations, only the final state could be evaluated and no direct comparison between different shapes and volumes could be performed in the same patient [5,8,18,19]. This problem was first solved in a study of Hidalgo and Weinstein, in which the same sized anatomical and round breast implants were used intraoperatively, and photos were taken in a sitting position [9]. However, two-dimensional representations are not representative. Moreover, the human eye is limited in its ability to detect topographic differences resulting from breast augmentation. In this study, we tried to create objective measures, and compare the topographic effect of breast implants of different shapes and sizes. Nevertheless, we must note some limitations, in part because of the minimal experience in applying the method presented.

First, in this interventional study, only ten patients (in total *n* = 130 breast scans) were included, so no statement could be made about the influence of individual differences such as the quality of the connective, muscular, and glandular tissue, nor the skin elasticity on the breast topography. Moreover, it is important to consider that the breast implant market is huge and there are many companies producing implants of different shapes, materials, and consistencies. In our study, only textured implants from a single company were used. Additionally, the pocket shape, the plane of the implant placement, and the incisional approach were arbitrarily chosen by the surgeon, which may have led to individual differences in postoperative results.

Our results show that the jugulum-nipple distance had a maximal variability of 3.1 mm throughout all SIs. No constant increase was observed with increasing volume in anatomical implants, with SI 6 resulting in a smaller jugulum-nipple distance compared to SI 4. This can be explained by the fact that with increasing augmentation, the nipple migrated into quadrant II in approximately 90% of the cases, thus leading to no marked increase in jugulum-nipple distance with increasing volume. As expected, the nipple-IMF distance increased with bigger implant sizes. Comparing between round and anatomical implants of the largest size (SI 5 vs. 6), a maximal difference of 2.8 mm in nipple-IMF distance was recorded. This supports the hypothesis that there is no notable difference in volume increase in the lower quadrants between round and anatomical implants. Nevertheless, the use of anatomical landmarks to describe topographic changes is not always accurate. Importantly, not all landmarks have an exact definition and depend on the shape and size of the anatomical region being described (e.g., IMF, lateral and medial breast fold). The lack of clear landmark definition and reproducibility has been discussed in previous studies [3,16,23,24].

As mentioned above, the nipple migrated into quadrant II in almost 90% of the cases regardless of implant shape or size. To date, there is barely any literature on the interaction between implants of different sizes and shapes and position of the nipple. In this study, it was possible to deliver objective data on topographic shift of the nipple, but only a rough direction of nipple migration was indicated. A method using a fixed coordinate system would allow for a more precise description.

The essential finding that round and anatomical implants have neither significant effect on the topography of single breast quadrants, nor on the entire topography of the breast, is concordant with findings in previous studies on the subjective perception of these effects [7,8,9,19,25]. The volumetric impact of all breast implants was mainly in quadrant II, followed by I, III, and IV. Hence, the volumetric increase due to anatomical implants could not principally be found in the lower quadrants, as it has been postulated in previous studies [20,21,22,26]. In addition, round implants have a volume increase of only 1% higher in quadrant I and 2% higher in quadrant II compared to anatomical implants, which is a difference not detectable by the human eye. Thus, the hypothesis that anatomical implants provide a more “natural” appearance can be rejected, as there is no evident difference in external projections between the two implant types, as was confirmed by a previous MRI study of Hamas et al. [18]. Nevertheless, it is important to note that our measurements were carried out intraoperatively, and because the breast shape usually changes in the postoperative months [1], no statement on long-term results can be made. Moreover, it is not possible to conclude that there is no significant difference in the augmented breast’s topography comparing round and anatomical implants with regards to the pocket preparation level and soft tissue coverage. In this study, a very young and not pre-operated patient population was analyzed. In the case of a thin soft tissue cover (e.g., after subcutaneous mastectomy), it might be necessary to select the implants more individually, as in these cases the implant shape is probably more dominant on the breast’s shape. In this study, implant shape did not lead to a significant topographic shift in the breasts of the analyzed patients. Other factors such as pocket preparation and surgical technique might be more decisive for the long-term breast contour. 

3D-simulations about postoperative results are often used preoperatively in the decision-making process of choosing the right implant. However, these 3D-models are frequently not based on objective measurements [27]. Although 2D image-based software has been proposed for instance by Cardoso et al. and Longo et al. as an objective form of preoperative breast volume assessment and as potential breast cosmesis tools that could be used in clinical trials and in surgical evaluation [28,29], we aimed to increase the accuracy of such software by adding another dimension. 3D-models allow for an objective representation of breast topography from different perspectives and could be included in such software and apps.

## 5. Conclusions

Implant size and not shape (round vs. anatomical) was decisive for the topographic change of the breast when it comes to breast augmentation. The volumetric effect of both round and anatomical implants was mainly located in the upper breast quadrants. No evident differences in distances between anatomical landmarks were observed comparing between round and anatomical implants of different sizes. Furthermore, nipple migration into quadrant II was seen in about 90% of augmentations.

## Figures and Tables

**Table 1:** Demographics. An overview of age, weight, BMI, and degree of ptosis for each patient of the study is provided. Data are presented as median (range).

Patient No.	Age (Years)	Weight (kg)	BMI (kg/m^2^)	Regnault’s Degree of Ptosis
1	24	59	22	0
2	23	76	25	0
3	31	75	26	2
4	49	58	22	1
5	22	68	24	0
6	47	60	22	1
7	27	54	22	2
8	46	65	25	0
9	33	60	19	1
10	32	69	20	0
Median (range)	32 (22–49)	63 (54–76)	22 (19–26)	0.5 (0–2)

**Table 2:** Volumes of the study implants (SI).

		SI 1	SI 2	SI 3	SI 4	SI 5	SI 6
	1	165.0	170.0	210.0	210.0	250.0	-
	2	295.0	290.0	400.0	390.0	505.0	495.0
	3	320.0	320.0	400.0	395.0	485.0	480.0
	4	165.0	165.0	220.0	220.0	275.0	275.0
Patient, No.	5	235.0	235.0	320.0	320.0	400.0	395.0
	6	165.0	165.0	220.0	220.0	-	-
	7	-	-	220.0	220.0	275.0	275.0
	8	185.0	185.0	255.0	255.0	315.0	315.0
	9	215.0	210.0	285.0	280.0	360.0	350.0
	10	235.0	235.0	320.0	320.0	400.0	395.0
N valid (missing)		9 (1)	9 (1)	10 (0)	10 (0)	9 (1)	8 (2)
Mean (cc)		220.0	219.4	285.0	283.0	362.8	372.5
SD (cc)		57.4	56.1	73.1	70.5	92.4	84.9
Minimum (cc)		165.0	165.0	210.0	210.0	250.0	275.0
Maximum (cc)		320.0	320.0	400.0	395.0	505.0	495.0

SI 1: round, small volume; SI 2: anatomical, small size; SI 3: round, medium size; SI 4: anatomical, medium size; SI 5: round, large size; SI 6: anatomical, large size. The volumes of the study implants are listed for each patient (no. 1–10). The number of included and missing implants is listed. The minimum, maximum, mean, and standard deviation (SD) of the volume of the SI are provided.

**Table 3:** Impact of main effects on topographic shift.

			95% CI		
		Estimated Mean (mm)	Lower Limit (mm)	Upper Limit (mm)	*p* Value
Shape	Round	6.56	5.47	7.66	0.90
	Anatomical	6.60	5.51	7.70	
Size	Small	5.31	4.17	6.45	
	Medium	6.55	5.43	7.67	<0.001
	Large	7.89	6.74	9.03	<0.001
Quadrant	I	8.08	6.91	9.25	
	II	9.16	7.99	10.33	<0.014
	III	6.96	5.79	8.13	<0.011
	IV	2.14	0.97	3.30	<0.001

The topographic shift (mm) caused by the main effects shape, size and quadrants is listed for each subcategory. Data are presented as mean and 95% confidence interval (CI), including lower and upper limit. The *p* value describes significant differences in topographic shift among subcategories of the main effects. Significance level is *p* ≤ 0.05.

**Table 4:** Inter-landmark distances. Nipple—jugulum, nipple—inframammary fold (IMF), nipple—medial breast fold and nipple—lateral breast fold distances are presented for each study implant (SI). Data are presented as mean ± standard deviation.

	Nipple—Jugulum (mm)	Nipple—IMF (mm)	Nipple—Medial Breast Fold (mm)	Nipple—Lateral Breast Fold (mm)
Native	188.3 ± 13.7	64.7 ± 10.0	87.2 ± 11.7	97.7 ± 8.8
SI 1	188.9 ± 12.3	85.8 ± 6.9	106.8 ± 7.0	116.5 ± 9.7
SI 2	189.5 ± 12.9	84.8 ± 6.8	107.5 ± 6.8	115.5 ± 8.4
SI 3	188.9 ± 12.7	90.5 ± 9.4	111.5 ± 6.4	121.3 ± 10.5
SI 4	188.0 ± 12.6	91.2 ± 9.2	111.5 ± 5.7	121.0 ± 10.8
SI 5	190.5 ± 13.9	99.1 ± 12.6	115.3 ± 6.4	131.1 ± 12.5
SI 6	187.4 ± 13.9	101.9 ± 11.6	116.3 ± 5.4	131.0 ± 11.3

**Table 5:** Nipple position shift. Q1 = superolateral quadrant, Q2 = superomedial quadrant, Q3 = inferomedial quadrant.

		Anatomical, No.			Total
		Q1	Q2	Q3	
Round, No.	Q1	2	1	0	3
	Q2	2	46	1	49
	Q3	1	1	0	2
Total		5	48	1	54

## Data Availability

The datasets used and/or analyzed during the current study are available from the corresponding author on reasonable request.
